# Atypical Presentation of Painless Herpes Zoster in an Elderly Male: A Case Report

**DOI:** 10.7759/cureus.75599

**Published:** 2024-12-12

**Authors:** Salim Barakat, Razan Dankar, Mohammad Aldalahmeh, Mohammad Barakat, Neville Mobarakai

**Affiliations:** 1 Internal Medicine, Staten Island University Hospital, Staten Island, USA; 2 Medicine, Lebanese University, Beirut, LBN; 3 Infectious Disease, Staten Island University Hospital, Staten Island, USA

**Keywords:** atypical rash, elderly individuals, shingles, shingle skin rash, varicella-zoster virus

## Abstract

Shingles, also known as herpes zoster, is a reactivation of the chickenpox virus that causes a painful, blistering rash. After a chickenpox infection, the virus lies dormant in nerve cells. When reactivated, usually in older adults or those with weakened immune systems, it travels along nerves, typically affecting a single strip of skin called a dermatome. Shingles usually presents as a painful, one-sided rash that may involve nerves in the head, face, and body. While it typically resolves on its own, it can lead to complications, especially in older adults. Antiviral medications are effective in reducing the severity and duration of shingles and should be started as soon as the rash appears. This report describes an unusual case of an elderly patient who developed shingles on their face after a wound, notably without experiencing any pain.

## Introduction

Herpes zoster (HZ) affects people globally year-round. Younger adults experience a lower incidence rate (1.2-3.4 cases per 1000 individuals annually) compared to elderly individuals over 65, who face a higher rate (3.9-11.8 cases per 1000 individuals annually) [1]. Studies from 2002 to 2018 estimate that roughly 3 to 20 out of every 1000 people will experience HZ, with females being more susceptible [2]. Major risk factors include age over 50, weakened immune systems, infections, mental stress, and diabetes, which increase the risk by approximately 38% according to a recent meta-analysis [3]. HZ occurs due to the reactivation of the dormant varicella-zoster virus (VZV), which remains inactive in the sensory nerve ganglia after initial exposure, usually in early childhood. HZ is typically marked by the appearance of a vesicular rash confined to a specific dermatomal area and is usually accompanied by severe neuralgic pain. These characteristic signs generally enable clinicians to diagnose HZ based solely on clinical observations [4].

In addition to skin symptoms, VZV reactivation can lead to several less common but serious complications, including various neurological issues, eye and organ disorders, as well as vascular problems. Diagnosing these complications can be straightforward when the typical zoster rash is present [5]. However, when the rash is absent or presents atypically, it can create uncertainty in diagnosis, delay the start of treatment, and result in extended morbidity [6]. Here, we report a case of an elderly patient presenting with an atypical, painless right facial rash, later diagnosed as a zoster infection.

## Case presentation

An 85-year-old male with a medical history of hypertension, hyperlipidemia, chronic atrial fibrillation, and right facial basal cell carcinoma post-Mohs surgery and skin graft presented to the emergency department (ED) with swelling and rash on the right side of his face. The rash was non-painful and had started one week prior after he developed a small cut while shaving his beard, with swelling and vesicles developing two days later. The patient was initially evaluated at urgent care, diagnosed with cellulitis, prescribed Keflex (cephalexin), and referred to the hospital for further evaluation and treatment.

**Figure 1:**
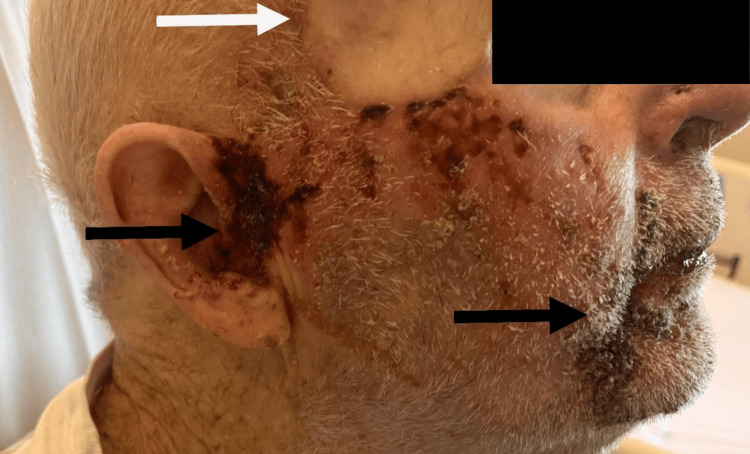
Right facial rash, preauricular necrotic tissue, perioral yellow and brown crusting (black arrows), intact skin graft (white arrow).

**Figure 2:**
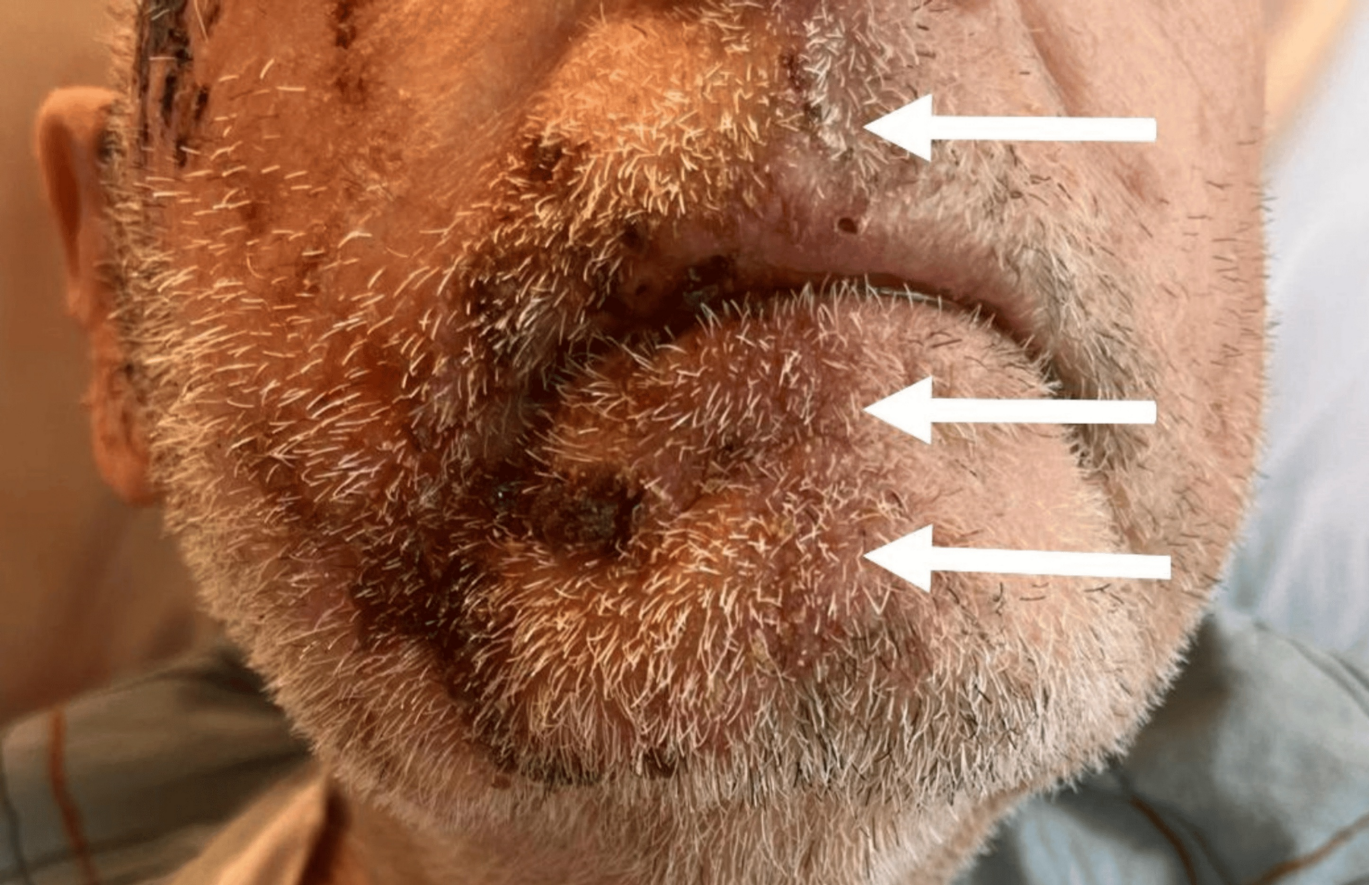
Right-sided perioral rash; rash does not cross the midline (white arrows).

Upon arrival at the ED, the patient was afebrile and was vitally stable. The physical examination revealed that the heart, lungs, abdomen, and body skin were normal. However, there was significant swelling accompanied by a rash and papules, as well as a 2 x 3 cm superficial wound in the right preauricular area, which exhibited notable necrotic tissue. Adherent yellow and brown crusting was also observed at the right cheekbone and peri-orally (Figures 1-2). Notably, the rash was confined to the right side of the face (Figure 2). There was no tenderness to palpation, induration, fluctuation, active bleeding, drainage, or malodor. The right temporal area had a well-healed and non-affected skin graft tissue (Figure 1).

**Figure 3:**
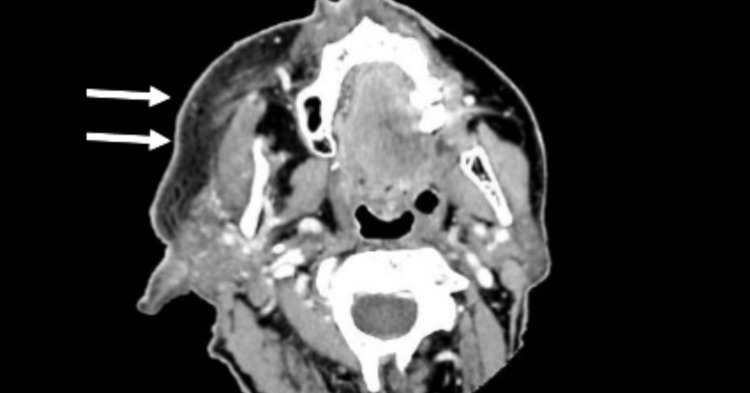
Maxillofacial CT scan: asymmetric subcutaneous edema and mild inflammation throughout the right side of the face (white arrows). No abscess.

Laboratory testing revealed a normal white blood cell count of 5900 cells per microliter of blood and an elevated C-reactive protein (CRP) of 27.6 mg/L. Additional infectious workup, which was collected on ED presentation, including blood and urine cultures, came back negative. A computed tomography (CT) scan with intravenous contrast of the maxillofacial area was significant for asymmetric subcutaneous edema and mild inflammation extending from the chin to the external auditory canal with no evidence of abscess (Figure 3).

In light of the findings, the patient was admitted with a working diagnosis of cellulitis and was started on intravenous Unasyn (ampicillin/sulbactam) and linezolid. The plastic surgery team was consulted and proceeded with local debridement of the necrotic wound. Tissue cultures were collected for microbiologic testing.

On the second day of admission, it was observed that some of the papules had progressed into vesicles. The infectious disease (ID) team was consulted for suspicion of VZV. VZV polymerase chain reaction (PCR) was obtained, and the patient was started on oral Valacyclovir, which was resumed after PCR results confirmed a diagnosis of zoster. The previously collected tissue cultures came back positive for *Staphylococcus haemolyticus* and *Staphylococcus epidermidis*, with the antibiogram showing pan sensitivity for the bacteria. On the fourth day of hospitalization, the patient’s rash showed significant improvement, and he was discharged on oral linezolid and valacyclovir to complete a total of 10 days of therapy.

## Discussion

The VZV, also known as human herpesvirus 3, is a neurotropic alphaherpesvirus responsible for causing chickenpox (varicella) and shingles (HZ) [7]. Shingles typically presents as a painful rash resulting from the reactivation of latent VZV and affects approximately one million people in the United States annually. Nearly one in three individuals will experience shingles in their lifetime [8].

HZ can be triggered by several factors, including age, immunosuppression, and stress [9]. Interestingly, trauma or injury, as in the case of our patient, has been reported as a potential trigger of VZV reactivation in several case reports [10,11]. For instance, Häfelfinger et al. described an 81-year-old patient who developed shingles after a minor forehead injury [10], while White et al. reported a 42-year-old woman who developed symptoms following a facial treatment with minimal injury [11]. These cases highlight the importance of considering physical trauma as a potential precipitating factor for HZ, especially when cellulitis or infection is coexisting, as in our patient.

Clinically, HZ typically progresses through three stages: pre-eruptive, acute eruptive, and chronic. The pre-eruptive stage usually involves burning or pain in the affected dermatome, occurring at least two days before the rash appears. Systemic symptoms such as headaches, malaise, and photophobia may also occur. During the acute eruptive phase, painful vesicles appear, which can later burst, ulcerate, and dry out. This phase is highly contagious and may last two to four weeks, with pain often persisting for a longer duration [12,13].

What makes our case particularly noteworthy is the atypical presentation of painless shingles. The complete absence of preherpetic or postherpetic neuralgia in our elderly patient is a rare occurrence, as pain is typically a hallmark symptom of HZ [14]. This atypical presentation, combined with the presence of cellulitis, led to a delay in diagnosis. While there are limited reported cases of painless HZ throughout the literature [15-18], our case underscores the importance of maintaining a high index of suspicion for atypical presentations, especially in elderly patients.

Several mechanisms may contribute to this atypical manifestation. Extensive damage to sensory neurons in the dorsal root ganglia during viral reactivation could disrupt pain signaling pathways [19]. In some cases, severe deafferentation of the affected skin area may result in a loss of pain sensation, potentially leading to painless scratching injuries [20]. Age-related changes, including decreased density of nerve fibers in the skin, might also reduce pain perception in elderly patients [19]. Additionally, the location of the affected dermatome may also influence pain presentation, with certain areas potentially having different pain sensitivities [20].

Early and accurate diagnosis is essential for effective management and reducing complications. Clinicians typically rely on clinical evaluation, but further testing through PCR and direct fluorescent antibody testing can confirm VZV. However, diagnosis can be challenging due to variable and atypical presentations, as exemplified by our case [12].

Regarding management, the standard treatment for HZ includes antiviral medications such as acyclovir and its prodrugs, valacyclovir and brivudine. Early initiation of antiviral therapy is crucial, ideally within 72 hours of rash onset, to reduce the severity and duration of symptoms and to prevent complications such as postherpetic neuralgia [12]. Valacyclovir is particularly beneficial due to its higher bioavailability, allowing for more effective viral suppression with less frequent dosing compared to acyclovir. In addition to antivirals, supportive care is important. While pain management is typically a key component of HZ treatment, including analgesics such as acetaminophen or nonsteroidal anti-inflammatory drugs (NSAIDs), and in severe cases, opioids or gabapentin [12,19], our patient's painless presentation negated this aspect of care.

## Conclusions

In conclusion, this case report of painless shingles in an elderly patient highlights the diverse clinical presentations of HZ. It emphasizes the need for clinicians to be aware of atypical manifestations, particularly in the context of recent trauma or coexisting infections. Understanding these varied presentations is crucial for effective diagnosis and management, especially in populations at increased risk, such as the elderly. Further studies are needed to explore the mechanisms behind painless zoster and to determine whether early antiviral therapy can prevent complications in these atypical cases. Clinicians should maintain a high index of suspicion for HZ in elderly patients, even if pain is not present. This vigilance is essential for timely diagnosis and appropriate management, potentially improving outcomes in this vulnerable population. By considering the possibility of atypical presentations, healthcare providers can ensure that cases of painless zoster are not overlooked, leading to prompt treatment and reduced risk of complications.
